# Edge-aware spatial-frequency extrapolation for consecutive block loss

**DOI:** 10.1186/s40064-016-2213-6

**Published:** 2016-05-11

**Authors:** Hao Liu, Dengcheng Wang, Bing Wang, Kangda Li, Hainie Tang

**Affiliations:** College of Information Science and Technology, Donghua University, Shanghai, 201620 China; Engineering Research Center of Digitized Textile & Fashion Technology, Ministry of Education, Donghua University, Shanghai, 201620 China

**Keywords:** Spatial error concealment, Consecutive block loss, Edge synthesis, Parametric model

## Abstract

To improve the spatial error concealment (SEC) for consecutive block loss, an edge-aware spatial-frequency extrapolation (ESFE) algorithm and its edge-guided parametric model are proposed by selectively incorporating the Hough-based edge synthesis into the frequency-based extrapolation architecture. The dominant edges that cross the missing blocks are firstly identified by the Canny detector, and then the robust Hough transformation is utilized to systematically connect these discontinuous edges. During the generation of edge-guided parametric model, the synthesized edges are utilized to divide the missing blocks into the structure-preserving regions, and thus the residual error is reliably reduced. By successively minimizing the weighted residual error and updating the parametric model, the known samples are approximated by a set of basis functions which are distributed in a region containing both known and unknown samples. Compared with other state-of-the-art SEC algorithms, experimental results show that the proposed ESFE algorithm can achieve better reconstruction quality for consecutive block loss while keeping relatively moderate computational complexity.

## Background

To reduce computational complexity and memory burdens, the block-based image/video coding has been widely used in visual communication. The transmission of coding data may lead to block losses in packet-switching networks. As a result, the decoded image may be greatly degraded. As a post-processing technique, error concealment (EC) is used to recover the missing blocks of an image by exploiting the spatial or temporal correlations (Usman et al. 2015). Although the temporal correlation tends to be higher than the spatial one, there are some situations where it is difficult to access temporal information, such as images and intra-coded frames (intra-frame). Under such circumstances, spatial error concealment (SEC) tries to reconstruct the damaged blocks by utilizing spatially neighboring pixels. Flexible macroblock ordering (FMO) can provide a common benchmark for comparing different SEC algorithms, where a frame is divided into several independently-decodable block groups, and each block group consists of a sequence of blocks (Panyavaraporn and Aramvith 2011). With FMO, different block groups are encapsulated into different packets, so that the missing blocks in the decoded image can be concealed by neighboring pixels. In some applications, the loss of one packet implies the loss of a block group within a frame.

The typical SEC algorithms mainly depend on the efficient use of spatial correlation. The Markov random field (MRF) is used as a prior model of natural images, and its model parameters are locally adjusted according to the image characteristics around the damaged region (Shirani et al. 1999). The MRF algorithm can produce a visually comfortable but sometimes over-smoothed concealment without a substantial increase in computational complexity. Bilinear interpolation (BI) is a well-known SEC technique as a non-normative part in block coding standards (Varsa et al. 2001), which uses the weighted averaging interpolation of neighboring pixels at vertical or horizontal boundaries of a damaged block. The BI algorithm can recover the smooth area but fail to restore the important edge information. To overcome this problem, it becomes necessary to rely on regularization techniques which go from simple low-pass filtering to more sophisticated edge-enhancement solutions (Cafforio et al. 2001). Current research on the problem mainly concentrates its efforts on the trade-offs between efficiency and accuracy.

Since the edge structures are visually more important than uniform textures, some advanced interpolation methods can exploit the structural information in the neighborhood of missing blocks. Li and Orchard (2002) proposed an orientation adaptive interpolation (OAI) algorithm based on a pixel-wise sequential prediction model, which estimates the missing block from eight directions in raster scan order and merges them with the weighted combination. The OAI algorithm alleviates error propagation at the expense of blurred details. In addition, the content-adaptive error concealment (CAEC) (Zhang et al. 2004) classifies each missing block into one of three categories: edge block, texture block and uniform block, and then conceals the missing blocks by different interpolation methods. By a minimum mean square error (MMSE) estimator, the probability function may be used to recovery the missing blocks. Koloda et al (2014a) suggested the MMSE-based error concealment with kernel density estimation (KMMSE), which need more computational complexity to improve reconstruction quality.

Adaptive predictors have been widely researched in lossless coding. If these predictors are directly utilized for SEC, they may cause the severe error propagation. Liu et al (2014) proposed an order-adaptive linear predictor (OALP) to sequentially estimate the missing pixels, where Bayesian information criterion is adopted to explicitly determine the order of the predictor, and error propagation can be well alleviated by a carefully designed scan order. As a typical technique for object removal applications, inpainting is also applicable to the SEC problem. With the loss of Shannon entropy, the inpainting-based SEC has an implicit advantage in terms of subjective evaluation. Since the image inpainting would require larger amount of computations, it is often difficult to be applied for the SEC applications with run-time constraints. Chung and Yim (2014). proposed a hybrid exemplar-based inpainting and spatial interpolation (HEISI) method, whose unique feature is the threshold-selective reconstruction by inpainting or interpolation. When there is a similar patch, HEISI performs the exemplar-based inpainting; otherwise, it performs the spatial interpolation. The edge synthesis is also used in spatial error concealment, where multi-directional interpolations are combined according to the visual clearness (VC) of the edges (Koloda et al. 2013). However, the VC algorithm is effective only when four neighboring blocks of a missing block are available, and it does not work for consecutive block loss.

The aforementioned SEC algorithms can effectively utilize the multi-directional correlation to combat the scattered block loss. When the consecutive block loss occurs, these SEC algorithms will face lots of difficulties to obtain any horizontal correlation, so the high-quality concealment has to rely on much prior knowledge (Usman et al. 2015). In this paper, our work concentrates on the error concealment of consecutive block loss, which is a more challenging scenario where multiple interleaved rows of blocks are missing. An alternative SEC approach is the signal extrapolation (Kaup et al. 2005; Koloda et al. 2014b), which can estimate the unknown signal parts from known samples by assuming that image signals can be sparsely represented in the frequency domain. Based on the successive approximation of parametric model, Koloda et al (2014b) proposed a modified frequency selective extrapolation (XFSE) algorithm that exploits the prior knowledge regarding the low-pass behavior of natural images, and yields a certain smoothing gains for consecutive block loss. However, due to high-frequency decaying of low-pass filter, XFSE cannot progressively improve its reconstruction quality even with more basis functions, whose performance saturates as the number of iterations is further increased. If high-frequency edge information is available, the low-pass filtering module in XFSE should be removed during the generation of parametric model, and thus the concealment performance is likely to be further improved.

The existing extrapolation algorithms don’t fully take into account the edge information of natural images. To further improve the reconstruction performance in case of consecutive block loss, we propose an edge-aware spatial-frequency extrapolation (ESFE) algorithm with its edge-guided parametric model, which incorporates the edge synthesis into the frequency-based extrapolation, and then exploits high frequency terms in image description. The ESFE algorithm firstly performs the segmentation to identify a plausible area of dominant edges, and then conceals the edge pixels across the missing blocks. The ESFE algorithm develops the edge-guided parametric model from the set of Fourier basis functions which can be used to replace the unknown samples with a low computational burden. For consecutive block loss, the ESFE algorithm utilizes the edge-guided parametric model to select optimal basis functions and expansion coefficients while preserving the edge information, and offers a much better solution in terms of reconstruction quality and complexity. To the best of our knowledge, this is the first study aiming to takes the edge information of consecutive block losses into account during the block-based concealment extrapolation.

The rest of this paper is organized as follows. “Problem formulation” section discusses the SEC problem with consecutive block loss, and provides a short review of signal extrapolation. Our proposed algorithm is described in “Proposed algorithm and its model” section. Extensive experimental results and performance comparisons are presented in “Experimental results” section. Finally, we conclude the paper in “Conclusions” section.

## Problem formulation

### Consecutive block loss

**Fig. 1:**
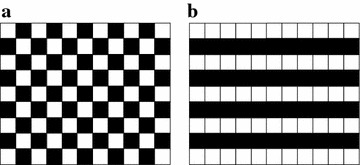
Two typical loss patterns. White squares are the correctly received blocks, while black squares are the missing blocks. **a** Scattered loss pattern, **b** consecutive loss pattern

Different SEC algorithms are designed to estimate the missing blocks from correctly received blocks. The locations of missing blocks can be obtained at the decoder. Figure 1 illustrates two typical loss patterns, i.e., the scattered loss pattern and the consecutive loss pattern, where one image is encoded into two block groups and each square represents a block of pixels. When a block group is missing, the test images are subjected to approximately 50 % block loss. Figure 1a shows a common situation of scattered loss pattern, where four-connected surrounding blocks of a missing block are correctly received. For the scattered block loss, many SEC algorithms can perform very well as the missing blocks can be reconstructed by their surrounding blocks. Another belongs to the consecutive loss pattern, which is a more challenging pattern since adjacent blocks in one row are lost. Figure 1b shows an example of consecutive loss pattern. Due to the lack of adjacent blocks in each row, many SEC algorithms cannot effectively combat the consecutive block loss. The consecutive block loss is still an open problem for spatial error concealment.

### Signal extrapolation

During signal extrapolation, the fitting of unknown samples is subject to a limited number of known samples, which may be used to estimate the missing blocks. In an extrapolation area, (*x*, *y*) and (*k*, *l*) indicates the (row, column) index in spatial domain and frequency domain, respectively. The samples of known blocks are successively approximated through a parametric model *g*(*x*, *y*), and the missing blocks are extrapolated according to a minimum error criterion of weighted energy function. During extrapolation process, the parametric model is a weighted superposition of two-dimensional basis functions *φ*_*k*,*l*_(*x*, *y*) with expansion coefficients *c*_*k*,*l*_.

1 $$g\left( {x,y} \right) = \mathop \sum \limits_{{\left( {k,l} \right) \in \varvec{F}_{{\mathbf{b}}} }} c_{k,l} \cdot \varphi_{k,l} \left( {x,y} \right)$$

where $\varvec{F}_{{\mathbf{b}}}$ denotes the index set in frequency domain, and the number of available basis functions equals the number of samples in the extrapolation area. The extrapolation mechanism iteratively updates a parametric model based on a set of basis functions, in order to approximate the available parts of received image. As the same time, the missing parts of received image can be estimated by the parametric model. Since there is only one basis function added to the model in every iteration step, certain iterations are needed for generating the model. Human visual system is very sensitive to the image structure (e.g., the edge or corner), so the proposed algorithm emphasizes on the concealment of missing region with dominant edges.

## Proposed algorithm and its model

### Edge synthesis via Hough transformation

Since the consecutive block loss will result in lower reconstruction quality, it is a practical strategy to firstly recover the part information of dominant edges, and the strategy provides a relatively robust basis for the following model generation. In this paper, the edge synthesis via Hough transformation is operated before signal extrapolation, where the edge detection need be introduced firstly to provide a binary edge map. For this purpose, the Canny’s edge detector is chosen due to a good compromise between efficiency and complexity (Canny 1986). Compared with other detectors such as Sobel or Prewitt, the Canny detector is less sensible to noise, and the detected edges are clear. Around the missing blocks, dominant edge points are obtained by the Canny detector. To connect the broken edges, Hough transformation has been widely used as the edge-connection tool (Robie and Mersereau 2000; Gharavi and Gao 2008). The merging approach in Ref. 16 is rather tedious, especially for a large number of consecutive block losses. In this paper we have utilized a more straightforward approach to connect the dominant edges. Based on the binary edge map from the Canny detector, the Hough transformation can connect the separated segments by a collinear set of points, since each line can be expressed as

2 $$\rho = x_{i} \cdot { \cos }\,\theta + y_{i} \cdot sin\,\theta$$

where ${\theta }$ (slope) and *ρ* (offset) denote the Hough coordinate, and the collinear points (*x*_*i*_, *y*_*i*_) with *i* = 1,…,*K*, are transformed into *K* sinusoidal curves which intersect at the same Hough coordinate ($\rho, \theta$). Each point (*x*_*i*_, *y*_*i*_) is transformed into a discretized curve and the accumulator cells along this curve are incremented. Since the collinear edge points in the spatial domain would accumulate into the same cell in the Hough domain, a high peak in the accumulator array would indicate the existence of a straight line in a missing block row.

**Fig. 2:**
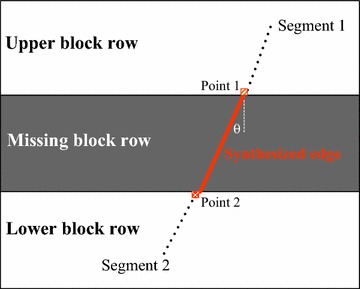
The synthesis illustration of a broken edge in a missing block row

For each Canny region which includes a missing block row, its upper block row, and its lower block row, a series of Hough procedures are implemented to acquire the dominant linear edges. The Hough transform is continuously applied to the binary regions provided by the Canny edge detector. Above and below a missing block row, two known segments with similar Hough coordinate are selected if their prolongation crosses the missing block row. There may be some near horizontal lines in the vicinity of the missing block row, which cannot be effectively used for edge synthesis. Some known segments with too large slope ${\theta }$ need be eliminated from the candidates. As shown in Fig. 2, if two known segments of a broken edge have similar (*ρ*, ${\theta }$) parameters, point 1 in the upper segment and point 2 in the lower segment respectively are the pixels which are closest to the missing block row. In the synthesized edge line, the gray level of a pixel is the bilinear interpolation between point 1 and point 2. After generating a binary edge, the broken edge is reconstructed by bilinear interpolation, whose missing pixel is replaced by a weighted mean of point 1 and point 2.

**Fig. 3:**
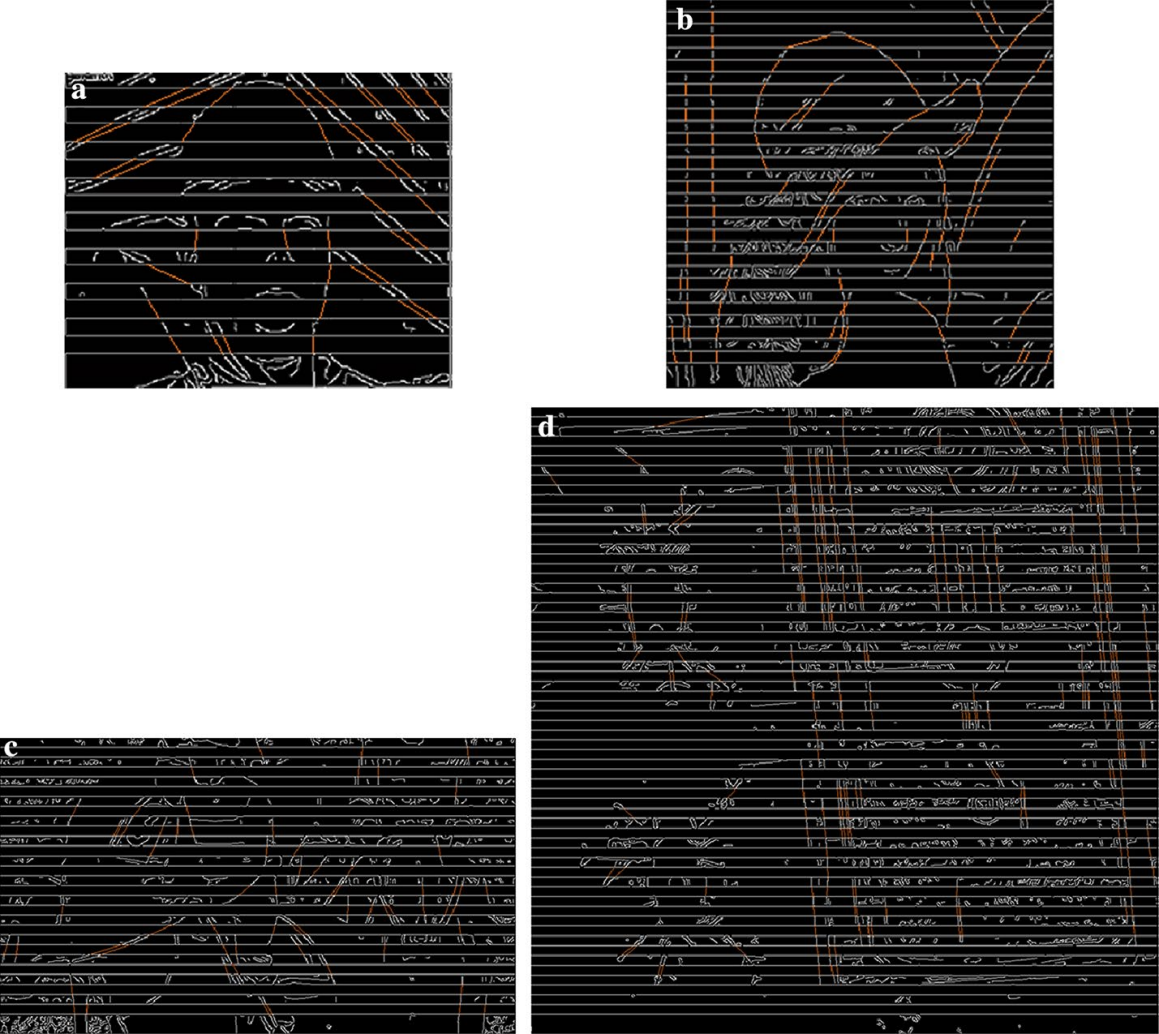
Experimental results of edge synthesis via Hough transformation, where yellow lines denote the synthesized edges. **a** Foreman (352 × 288, QP = 22), **b** Lena (512 × 512), **c** RaceHorses (832 × 480, QP = 37), **d** Airport (1024 × 1024)

In order to further clarify the mechanism of edge synthesis via Hough transformation, Fig. 3 shows the experimental results for four typical images and intra-frames: 1st intra-frame of *Foreman* (352 × 288, QP = 22), *Lena* (512 × 512), 1st intra-frame of *RaceHorses* (832 × 480, QP = 37), *Airport* (1024 × 1024), where the yellow line represents the synthesized edge among missing blocks, and the experimental settings are given in “Experimental results” section. These images and intra-frames have different resolutions, ranging from 352 × 288 to 1024 × 1024. It can be seen that the proposed mechanism can obtain basic structural information, and many dominant edges have been detected and connected successfully. The synthesized edges are then used to segment the blocks into different regions for the spatial-frequency extrapolation.

### Spatial-frequency extrapolation

**Fig. 4:**
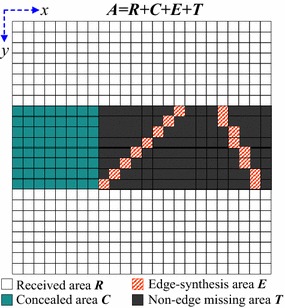
An extrapolation area $\varvec{A}$ as union of received area $\varvec{R}$, concealed area $\varvec{C}$, edge-synthesis area $\varvec{E}$, and non-edge missing area $\varvec{T}$

Based on the synthesized edge in “Edge synthesis via Hough transformation” section, the proposed algorithm will further improve the XFSE implementation. This approach is based on the XFSE method proposed by Koloda et al (2014b). After the dominant edges are found via the Hough transform, fine concealment can be achieved by spatial-frequency extrapolation. As illustrated in Fig. 4, the size of an extrapolation area $\varvec{A}$ is *X* × *Y* samples which are indexed by spatial variables *x* and *y.* All samples in the area $\varvec{A}$ belong to one of four areas: the correctly received samples build up the received area $\varvec{R}$; the samples from the missing blocks which have been extrapolated build up the concealed area $\varvec{C}$; in the current missing blocks, the synthesized edge samples build up the edge-synthesis area $\varvec{E}$, and other unknown samples such as texture belong to the non-edge missing area $\varvec{T}$. The following edge-guided parametric model is based on the extrapolation area in Fig. 4.

In order to reconstruct the unknown samples, we need to minimize a weighted residual error between the original signal and its parametric model. To estimate the samples in the non-edge missing area $\varvec{T}$, the parametric model successively approximates the available samples within the support area (the received area $\varvec{R}$, concealed area $\varvec{C}$, edge-synthesis area $\varvec{E}$). At the $n{\text{th}}$ iteration, the edge-guided parametric model *g*^(*n*)^(*x*, *y*) is:

3 $$g^{\left( n \right)} \left( {x,y} \right) = \mathop \sum \limits_{{\left( {p,q} \right) \in \varvec{F}_{{\mathbf{b}}} }} c_{p,q}^{\left( n \right)} \cdot \varphi_{p,q} \left( {x,y} \right)$$

where $\varvec{F}_{{\mathbf{b}}}$ denotes the set of basis functions $\varphi_{p,q} \left( {x,y} \right)$ weighted by the expansion coefficients *c*_*p*,*q*_^(*n*)^ at the $n{\text{th}}$ iteration; (*p*, *q*) denotes the (row, column) index in frequency domain. The samples in the support area are approximated successively by computing the basis function and expansion coefficient per iteration, where the basis function *φ*_*p*,*q*_(*x*, *y*) is selected which maximizes the decrease of residual error, and the expansion coefficient *c*_*p*,*q*_^(*n*)^ is computed by minimizing the residual error. The edge-guided parametric model is generated with the initialization *g*^(0)^(*x*, *y*) being 0, whose coefficients *c*_*u*,*v*_^(0)^ are also set to 0. At the $n{\text{th}}$ iteration, we can express the residual error as follows:

4 $$r^{\left( n \right)} \left( {x,y} \right) = \left( {s\left( {x,y} \right) - g^{\left( n \right)} \left( {x,y} \right)} \right) \cdot m\left( {x,y} \right)$$

where *s*(*x*, *y*) denotes a sample in the extrapolation area; the masking function *m*(*x*, *y*) is zero for (*x*, *y*)∈ $\varvec{T}$ and one otherwise, so as to ensure that the non-edge missing samples are not used. The residual error *r*^(*n*)^(*x*, *y*) between the available sample *s*(*x*, *y*) and the current model *g*^(*n*)^(*x*, *y*) is minimized. The edge synthesis can obtain some structural information in missing block rows, and thus reduce the residual error of edge-guided parametric model. The expansion coefficient is estimated by minimizing the weighted energy from the last residual error:

5 $$E_{p,q}^{{\left( {n + 1} \right)}} = \mathop \sum \limits_{{\left( {x,y} \right) \in \varvec{A}}} \omega \left( {x,y} \right)\left| {r^{\left( n \right)} \left( {x,y} \right)} \right|^{2}$$

where *ω*(*x*, *y*) is a weighting function. It has been demonstrated that the influence of the weighting function decays symmetrically with distance from the center of the missing block (Koloda et al. 2013). As a prior knowledge, the known sample in the vicinity of missing block has higher importance than the sample that is far from it, and the influence of the weighting function decreases with distance. Based on the synthesized edge in “Edge synthesis via Hough transformation” section, the proposed ESFE algorithm further refines the weighting function of XFSE by adding the edge-aware spatial correlation constraints. The new weighting function *ω*(*x*, *y*) can be defined as

6 $$\omega \left( {x,y} \right) = \left\{ {\begin{array}{*{20}c} {\beta^{{\sqrt {\left( {x - \frac{X - 1}{2}} \right)^{2} + \left( {y - \frac{Y - 1}{2}} \right)^{2} } }} } & {\forall \left( {x,y} \right) \in R} \\ {\varepsilon \cdot \beta^{{\sqrt {\left( {x - \frac{X - 1}{2}} \right)^{2} + \left( {y - \frac{Y - 1}{2}} \right)^{2} } }} } & {\forall \left( {x,y} \right) \in E} \\ {\delta \cdot \beta^{{\sqrt {\left( {x - \frac{X - 1}{2}} \right)^{2} + \left( {y - \frac{Y - 1}{2}} \right)^{2} } }} } & {\forall \left( {x,y} \right) \in C} \\ 0 & {\forall \left( {x,y} \right) \in T} \\ \end{array} } \right. .$$

During the spatial-frequency extrapolation, *ω*(*x*, *y*) is used for quantizing the influence of the distance to the extrapolated sample. As the unknown samples cannot contribute to the model generation, they have to be excluded from the weighting function, and the weight in the area $\varvec{T}$ is set to 0. The predetermined constant $\beta \in \left[ {0, 1} \right)$ controls the speed of the decaying. In the edge-synthesis area $\varvec{E}$, the influence for the sample is further weighted by a factor $\varepsilon \in \left( {0, 1} \right]$. In the concealed area $\varvec{C}$, the influence for the sample is further weighted by another factor $\delta \in \left( {0, 1} \right]$. At each iteration *n*, the projection variable of expansi coefficient can be expressed as

7 $$\Delta c_{p,q} = \frac{{\mathop \sum \limits_{{\left( {x,y} \right) \in \varvec{A}}} r^{\left( n \right)} \left( {x,y} \right) \cdot \varphi_{p,q}^{*} \left( {x,y} \right) \cdot \omega \left( {x,y} \right)}}{{\mathop \sum \limits_{{\left( {x,y} \right) \in \varvec{A}}} \varphi_{p,q}^{*} \left( {x,y} \right) \cdot \omega \left( {x,y} \right) \cdot \varphi_{p,q} \left( {x,y} \right)}}$$

which is interpreted as a weighted projection variable of *r*^(*n*)^(*x*, *y*) on *φ*_*p*,*q*_(*x*, *y*). The best basis function and its expansion coefficient need to be searched as the one which maximizes the reduction of error energy, that is,

8 $$\left( {u,v} \right) = \mathop {\text{argmax}}\limits_{{\left( {u,v} \right)}} \left\{ {\left| {\Delta c_{p,q} } \right|^{2} \mathop \sum \limits_{{\left( {x,y} \right) \in \varvec{A}}} \varphi_{p,q}^{*} \left( {x,y} \right) \cdot \omega \left( {x,y} \right) \cdot \varphi_{p,q} \left( {x,y} \right)} \right\}$$

The process of spatial-frequency extrapolation can be further described as follows:

1. Initializing the weighted residual error

After the edge-guided parametric model is generated, all the unknown samples are taken from the model, and inserted at the corresponding positions of missing samples. Let us consider the spatially-weighted version of the residual error. The parametric model is initialized by *g*^(0)^(*x*, *y*) = 0. The initialization {*n* = 0} of the weighted residual error is done by the following:

9 $$r_{\omega }^{\left( n \right)} \left( {x,y} \right) = \omega \left( {x,y} \right) \cdot r^{\left( n \right)} \left( {x,y} \right)$$

2. Determining the best fitting basis function

The Fourier basis can be selected arbitrarily so as to reflect the stochastic properties of an image. By using the two-dimensional discrete Fourier transform ($\varvec{DFT}$), the reduction of the weighted error energy can be expressed in the frequency domain:

10 $$\Delta E_{p,q}^{\left( n \right)} = \frac{{\left| {R_{\omega }^{\left( n \right)} \left( {p,q} \right)} \right|^{2} }}{{W\left( {0,0} \right)}}$$

where *R*_*ω*_^(*n*)^(*p*, *q*) and *W*(*p*, *q*) are the $\varvec{DFT}$ of *r*_*ω*_^(*n*)^(*x*, *y*) and *ω*(*x*, *y*), respectively. As the denominator in the equation above is constant, the division can be calculated in advance and be replaced by a multiplication with 1/*W*(0, 0) within the iteration loop.

3. Determining the expansion coefficients

Due to the inclusion of high-frequency edge information, the proposed ESFE algorithm further removes the low-pass filtering module of XFSE. Based on Eq. (7), the projection variable of expansion coefficient can be expressed in the frequency domain:

11 $$\Delta c_{u,v} = XY\frac{{R_{\omega }^{\left( n \right)} \left( {u,v} \right)}}{{W\left( {0,0} \right)}}$$

where *R*_*ω*_^(*n*)^(*u*, *v*) and *W*(*u*, *v*) are another $\varvec{DFT}$ of *r*_*ω*_^(*n*)^(*x*, *y*) and *ω*(*x*, *y*), respectively. The best basis function will be the one which can better approximate this residual error by the derivation of nearest neighbors. At the $\left( {n + 1} \right){\text{th}}$ iteration, the expansion coefficient *c*_*u*,*v*_^(*n*+1)^ is updated by

12 $$c_{u,v}^{{\left( {n + 1} \right)}} = c_{u,v}^{\left( n \right)} + {\gamma } \cdot \Delta c_{u,v}$$

where the compensation factor ${\gamma }$ is introduced to compensate the orthogonality deficiency of signal extrapolation. Smaller compensation factor yields a better convergence and slower quality decrease after a certain number of iterations.

4. Updating the edge-guided parametric model

The parametric model is updated at each iteration, which obtains optimal basis functions and expansion coefficients to successively approximate the available samples. The evolution of iterative procedure relies on the computation of weighted residual error *R*_*ω*_^(*n*)^(*p*, *q*). At the next iteration, the weighted residual error is updated as:

13 $$R_{\omega }^{{\left( {n + 1} \right)}} \left( {p,q} \right) = R_{\omega }^{\left( n \right)} \left( {p,q} \right) - \frac{1}{XY} \cdot \Delta c_{p,q} \cdot W\left( {p - u,q - v} \right)$$

where *R*_*ω*_^(*n*+1)^(*p*, *q*) are the $\varvec{DFT}$ of *r*_*ω*_^(*n*+1)^(*x*, *y*), which provides the weighted residual error for the next iteration directly in the frequency domain.

5. Final parametric model

After all iterations are done, the final parametric model is obtained by two-dimensional inverse discrete Fourier transform ($\varvec{IDFT}$). The unknown pixels are properly concealed from the edge-guided parametric model. This parametric model is the closest approximation to the known samples in the available support area:

14 $$g\left( {x,y} \right) = \varvec{IDFT}_{X,Y} \left[ {G\left( {p,q} \right)} \right]$$

## Experimental results

The proposed ESFE algorithm will be compared with other state-of-the-art SEC methods, such as MRF (Shirani et al. 1999), BI (Varsa et al. 2001), OAI (Li and Orchard 2002), CAEC (Zhang et al. 2004), KMMSE (Koloda et al. 2014a), OALP (Liu et al. 2014), HEISI (Chung and Yim 2014), and XFSE (Koloda et al. 2014b). Standard test images and video frames are examined on the consecutive loss pattern in Fig. 1b, which are subjected to about 50 % block loss. In order to facilitate the file operation, the last block row is retained. Peak signal-to-noise ratio (PSNR) is chosen as one of the objective quality metrics in the experiments, and the multi-scale structural similarity (MS-SSIM) metric is also reported (Wang et al. 2003). Most of previous SEC algorithms focus on the block losses with block size of 16 × 16 pixels. So each block has dimensions of 16 × 16 pixels and the size of the area $\varvec{A}$ is 48 × 48. Based on MATLAB 2012a, the Canny edge detector with adaptive threshold is utilized, and a series of Hough transform-based functions are applied to systematically connect the dominant edges. Typically, the weighting function declines with *β* = 0.8, and the orthogonality deficiency compensation is set to ${\gamma }$ = 0.2, and the parameters *ɛ* and *δ* are set to 1.0 and 0.9, respectively.

### Test on still images

To evaluate the performance of the proposed ESFE algorithm during image transmission, extensive experiments are conducted on several still images. The test images are selected from the USC-SIPI database (http://sipi.usc.edu/database), and they are uniformed into grayscale images of 8-bit depths. These test images include *Aerial* (256 × 256), *Peppers* (512 × 512), *Lena* (512 × 512), *Baboon* (512 × 512), *Boat* (512 × 512), *House* (512 × 512), *Airport* (1024 × 1024), and *Man* (1024 × 1024). When the consecutive block loss is applied in test images, the objective quality of different SEC algorithms is given in Table 1. As can be observed from the table, the proposed ESFE algorithm achieves the best average quality for all test images. The average gains over the second best algorithm are 0.48 dB in terms of PSNR and 0.0077 in terms of MS-SSIM. Moreover, our algorithm achieves up to 1.89 dB higher PSNR and 0.0701 higher MS-SSIM than the edge-directed OAI method. Compared with the recent HEISI, OALP, and XFSE methods, our algorithm averagely obtains gains of 0.48 dB, 0.89 dB, and 0.85 dB in terms of PSNR, and gains of 0.008, 0.019, and 0.0077 in terms of MS-SSIM. We attribute this remarkable improvement to the robust edge synthesis and well-designed signal extrapolation.

**Table 1:** Objective quality comparisons of different SEC algorithms for still images

	CAEC	BI	KMMSE	MRF	OAI	HEISI	OALP	XFSE	ESFE
*Aerial*									
PSNR (dB)	14.64	18.41	19.37	20.25	19.73	20.54	20.17	20.01	20.83
MS-SSIM	0.4542	0.7145	0.7481	0.7312	0.7626	0.7875	0.7643	0.7779	0.7862
*Peppers*									
PSNR (dB)	14.13	21.99	24.86	24.56	25.15	25.64	25.19	25.90	26.37
MS-SSIM	0.5212	0.8360	0.9358	0.9230	0.9101	0.9312	0.9260	0.9410	0.9478
*Lena*									
PSNR (dB)	15.37	22.87	27.63	27.05	26.51	27.95	27.21	27.62	28.68
MS-SSIM	0.5776	0.8293	0.9472	0.9326	0.9007	0.9474	0.9307	0.9383	0.9459
*Baboon*									
PSNR (dB)	14.69	20.63	21.99	22.17	20.44	22.96	22.68	22.20	23.29
MS-SSIM	0.3950	0.7547	0.8409	0.8110	0.6910	0.8395	0.8431	0.8408	0.8489
*Boat*									
PSNR (dB)	15.09	22.19	22.71	22.93	23.29	24.04	23.66	23.82	24.59
MS-SSIM	0.5864	0.8446	0.8590	0.8455	0.8152	0.8705	0.8533	0.8694	0.8813
*House*									
PSNR (dB)	12.81	21.53	22.23	23.14	22.56	23.53	23.21	22.76	23.61
MS-SSIM	0.4188	0.8278	0.8327	0.8435	0.8083	0.8417	0.8357	0.8477	0.8512
*Airport*									
PSNR (dB)	19.49	23.61	24.24	24.19	22.48	24.96	24.58	24.55	25.49
MS-SSIM	0.6381	0.8266	0.8682	0.8561	0.7596	0.8695	0.8530	0.8683	0.8767
*Man*									
PSNR (dB)	14.79	23.17	24.93	24.57	23.65	25.52	25.15	25.28	26.13
MS-SSIM	0.5352	0.8319	0.9069	0.8881	0.8393	0.8964	0.8896	0.9028	0.9103
*Average*									
PSNR (dB)	15.13	21.80	23.50	23.61	22.98	24.39	23.98	24.02	24.87
MS-SSIM	0.5158	0.8082	0.8674	0.8539	0.8109	0.8730	0.8620	0.8733	0.8810

**Fig. 5:**
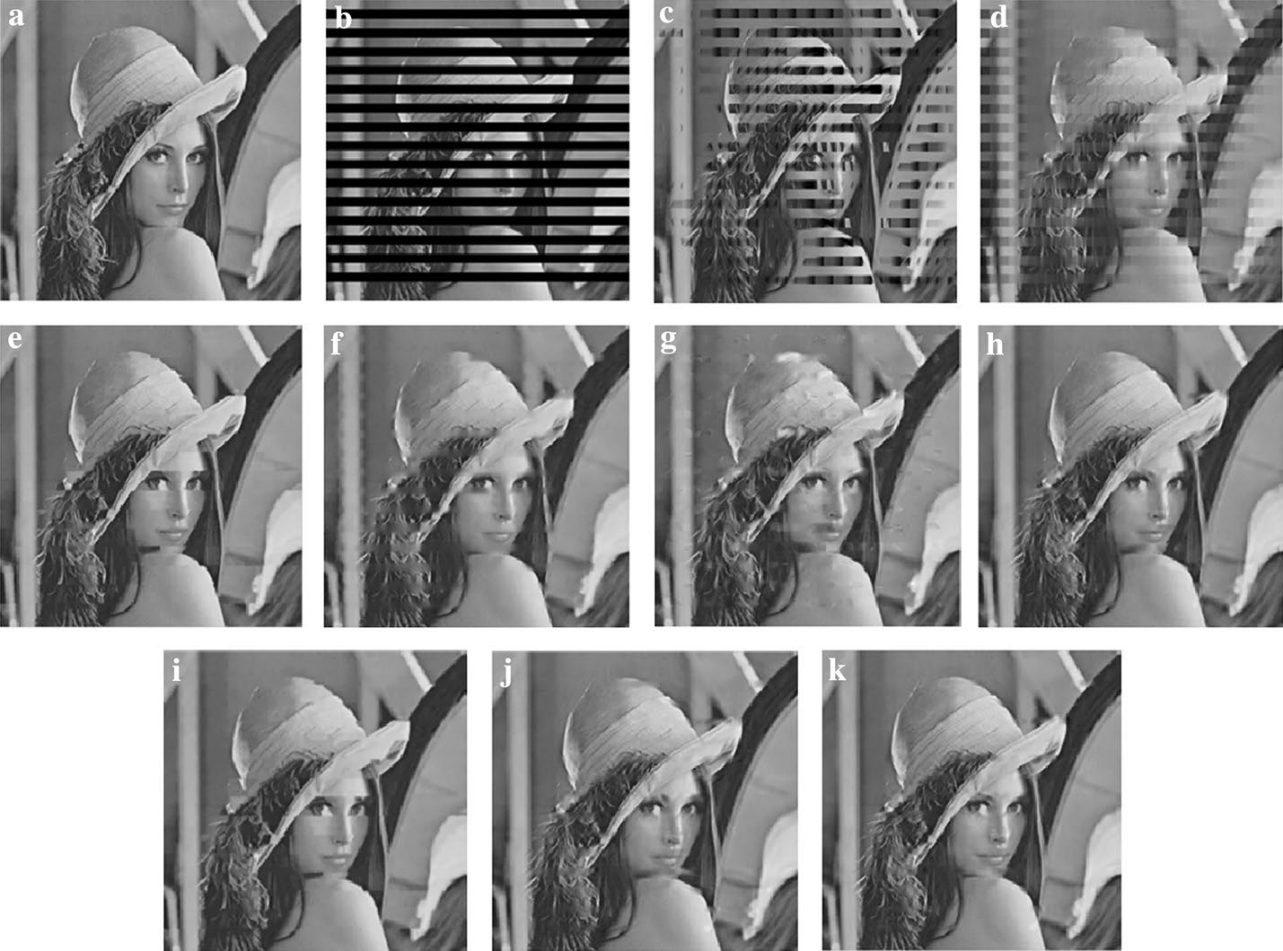
Subjective quality comparisons of different SEC algorithms for *Lena*. **a** The original image, **b** the corrupted image, **c** CAEC, **d** BI, **e** KMMSE, **f** MRF, **g** OAT, **h** HEISI, **i** OALP, **j** XFSE, **k** ESFE

**Fig. 6:**
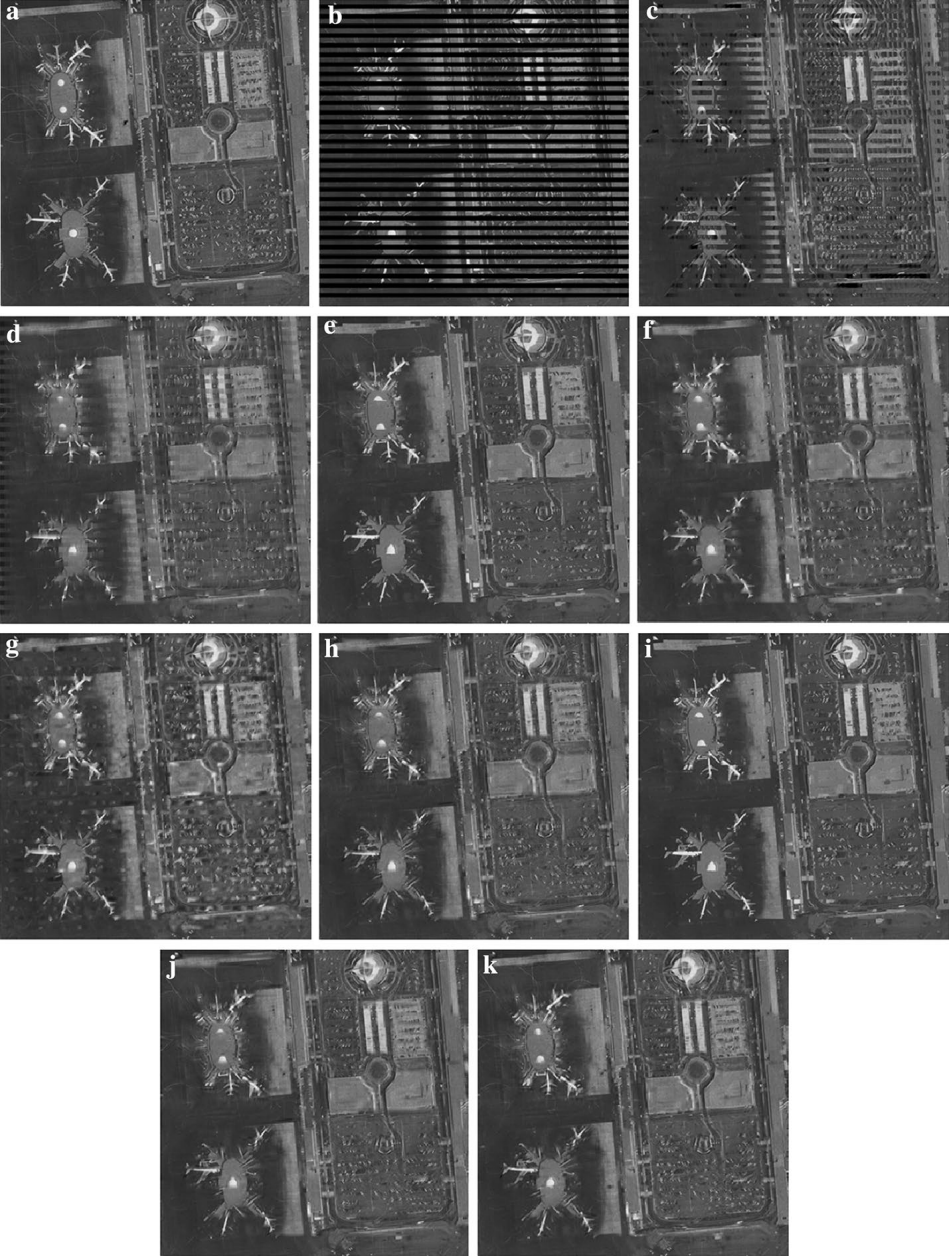
Subjective quality comparisons of different SEC algorithms for *Airport.* **a** The original image, **b** the corrupted image, **c** CAEC, **d** BI, **e** KMMSE, **f** MRF, **g** OAT, **h** HEISI, **i** OALP, **j** XFSE, **k** ESFE

For the corrupted images *Lena* and *Airport*, Figs. 5 and 6 show the subjective quality comparisons of reconstructed images by different SEC algorithms. From these figures in Fig. 5c, d, we can observe that the CAEC and BI algorithms face the difficulties to recover the edge information in the missing blocks, which result in severe blocking artifacts. In Fig. 5e–j, there are still many edges unrecovered, and they produce lumpy transition. In Fig. 5k, the proposed ESFE algorithm has recovered some dominant edges successfully, and noticeable improvements can be found around some regions (e.g., around the rim of hat and chin of *Lena*).

Figure 6 gives the similar conclusion. It can be observed that CAEC and BI methods completely blur the inner pixels of missing blocks, which heavily degrade visual quality. The OAI algorithm also produces some annoying artifacts. OALP and KMMSE produce some ghosting parts (e.g., around the head and wings of some airplanes). HEISI over-emphasizes the object contour and produces a few pseudo-edges. It is noticed that the proposed ESFE algorithm can more accurately recover global object contours with severe losses, such as the edges along the airport runway in the upper part of *Airport*.

### Test on intra-frames

The proposed ESFE algorithm is also utilized to recover the intra-frames to demonstrate its effectiveness when blocking artifacts and blurring caused by compression are present. Four standard sequences with different resolutions and different levels of activities are chosen: *Akiyo* (176 × 144), *Foreman* (352 × 288), *BlowingBubbles* (416 × 240), and *RaceHorses* (832 × 480). By H.264 reference software JM19.0 (Extended profile), 1st intra-frame of each sequence is encoded in grayscale. The entropy coding method is UVLC with only 4 × 4 transform. Without the rate control, the quantization parameter (QP) is set to 22, 27, 32, and 37, respectively. In the simulation, each frame is encoded into two block groups by using the consecutive loss pattern in Fig. 1b. Most parameters are default settings in the JM19.0 config file (Grayscale = 1, IDRPeriod = 1). Table 2 gives the objective quality comparisons of different SEC algorithms for intra-frame concealment with different QP values. It can be seen that the proposed ESFE algorithm can consistently improve the reconstruction quality over other SEC algorithms, and averagely outperforms them in terms of both PSNR and MS-SSIM. Compared to the second best algorithm, the average gains of ESFE are 0.77 dB in terms of PSNR and 0.0009 in terms of MS-SSIM.

**Table 2:** Objective quality comparisons of different algorithms for intra-frame concealment with different QP

Frames	QP		CAEC	BI	KMMSE	MRF	OAI	HEISI	OALP	XFSE	ESFE
*Akiyo* (176 × 144)	22	PSNR (dB)	20.21	22.55	25.33	25.75	23.90	25.88	25.56	25.23	26.29
		MS-SSIM	0.6931	0.8404	0.9022	0.8750	0.8265	0.9087	0.9034	0.8927	0.9043
	27	PSNR (dB)	19.71	22.02	24.73	25.12	23.32	25.26	24.95	24.62	25.63
		MS-SSIM	0.6691	0.8134	0.8740	0.8473	0.7998	0.8804	0.8751	0.8646	0.8760
	32	PSNR (dB)	19.29	21.54	24.21	24.61	22.83	24.73	24.43	24.11	25.13
		MS-SSIM	0.6551	0.7965	0.8557	0.8296	0.7831	0.8620	0.8569	0.8466	0.8577
	37	PSNR (dB)	18.47	20.63	23.19	23.57	21.87	23.69	23.40	23.09	24.07
		MS-SSIM	0.6271	0.7625	0.8193	0.7943	0.7497	0.8253	0.8204	0.8105	0.8212
*Foreman* (352 × 288)	22	PSNR (dB)	11.22	20.24	25.39	24.44	24.84	26.54	25.26	26.45	27.28
		MS-SSIM	0.4199	0.8240	0.9175	0.8711	0.8759	0.9185	0.9126	0.9170	0.9225
	27	PSNR (dB)	10.79	19.63	24.68	23.75	24.14	25.81	24.55	25.72	26.53
		MS-SSIM	0.3914	0.7873	0.8790	0.8335	0.8382	0.8802	0.8741	0.8785	0.8836
	32	PSNR (dB)	10.56	19.22	24.17	23.26	23.64	25.27	24.04	25.18	25.98
		MS-SSIM	0.3829	0.7704	0.8605	0.8159	0.8205	0.8614	0.8557	0.8601	0.8652
	37	PSNR (dB)	10.21	18.40	23.24	22.27	22.63	24.20	23.02	24.12	24.88
*BlowingBubbles* (416 × 240)		MS-SSIM	0.3659	0.7374	0.8234	0.7807	0.7851	0.8243	0.8188	0.8229	0.8279
	22	PSNR (dB)	17.54	22.88	23.86	23.84	23.19	23.58	23.47	24.11	24.47
		MS-SSIM	0.5900	0.8053	0.8617	0.8300	0.8065	0.8571	0.8529	0.8493	0.8596
	27	PSNR (dB)	16.69	21.92	22.87	22.86	22.23	22.61	22.50	23.12	23.48
		MS-SSIM	0.5281	0.7390	0.7943	0.7632	0.7402	0.7898	0.7857	0.7822	0.7923
	32	PSNR (dB)	16.33	21.45	22.40	22.38	21.75	22.13	22.02	22.63	22.98
		MS-SSIM	0.5162	0.7227	0.7769	0.7465	0.7239	0.7725	0.7684	0.7650	0.7749
	37	PSNR (dB)	15.63	20.53	21.43	21.41	20.81	21.18	21.08	21.66	22.03
*RaceHorses* (832 × 480)		MS-SSIM	0.4924	0.6902	0.7421	0.7129	0.6913	0.7379	0.7340	0.7307	0.7402
	22	PSNR (dB)	15.66	21.07	22.59	22.30	26.49	25.09	25.45	23.42	26.27
		MS-SSIM	0.5598	0.8152	0.8928	0.8618	0.7986	0.8484	0.8328	0.8857	0.8915
	27	PSNR (dB)	15.05	20.34	21.84	21.55	25.65	24.38	24.64	22.65	25.44
		MS-SSIM	0.5385	0.7687	0.8447	0.8144	0.7525	0.8013	0.7860	0.8378	0.8435
	32	PSNR (dB)	14.73	19.92	21.38	21.30	25.32	23.77	24.12	22.18	24.91
		MS-SSIM	0.5072	0.7522	0.8267	0.7970	0.7364	0.7842	0.7691	0.8199	0.8255
	37	PSNR (dB)	14.10	19.06	20.47	20.30	24.05	22.76	23.10	21.23	23.85
		MS-SSIM	0.4846	0.7193	0.7906	0.7622	0.7041	0.7499	0.7356	0.7841	0.7895
Average		PSNR (dB)	15.39	20.71	23.24	23.04	23.54	24.18	23.85	23.72	24.95
		MS-SSIM	0.5263	0.7715	0.8413	0.8085	0.7770	0.8314	0.8238	0.8342	0.8422

**Fig. 7:**
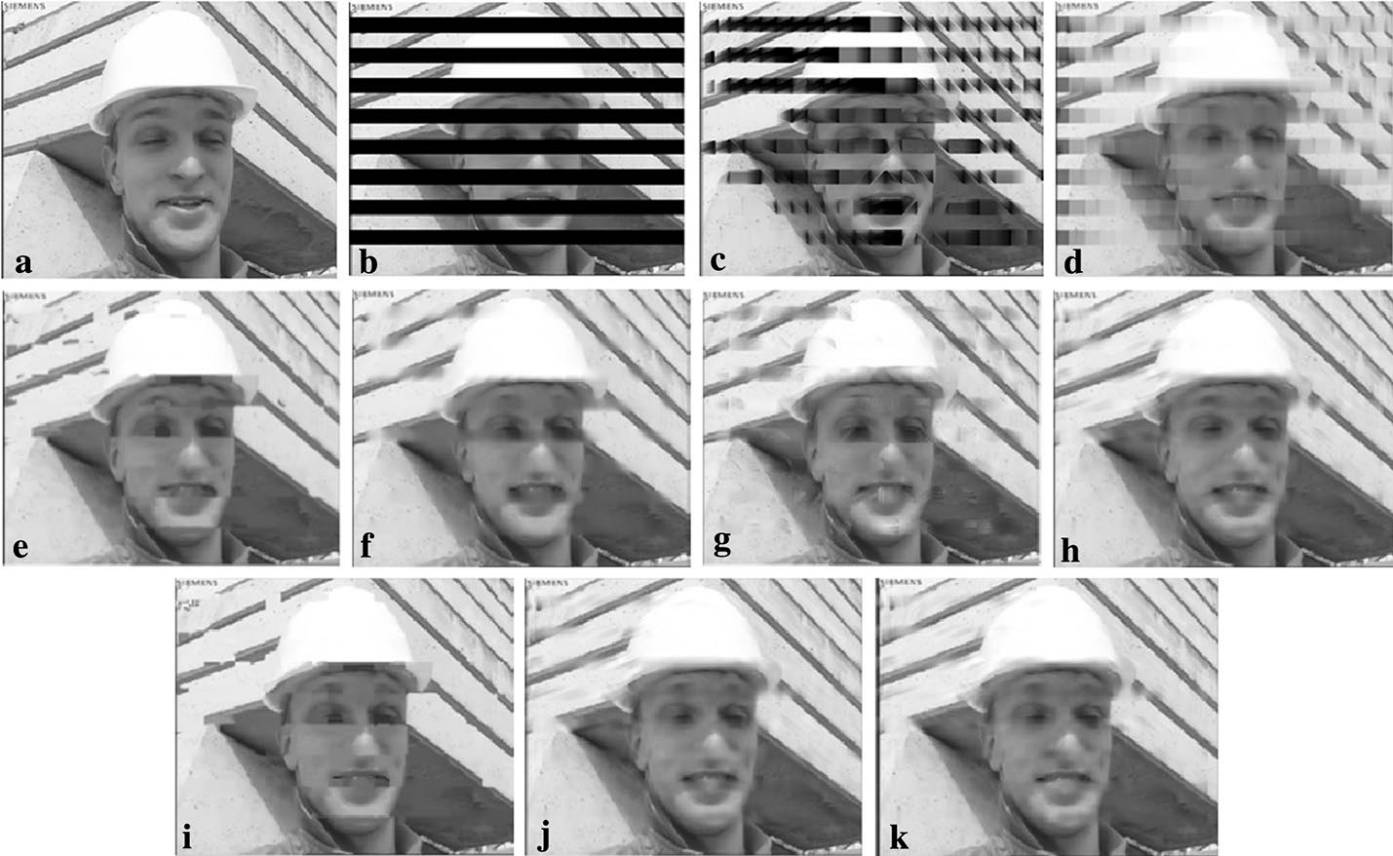
Subjective quality comparisons of different SEC algorithms for *Foreman*, 1st intra-frame in QP = 22. **a** The intra-coded frame, **b** the corrupted frame, **c** CAEC, **d** BI, **e** KMMSE, **f** MRF, **g** OAT, **h** HEISI, **i** OALP, **j** XFSE, **k** ESFE

**Fig. 8:**
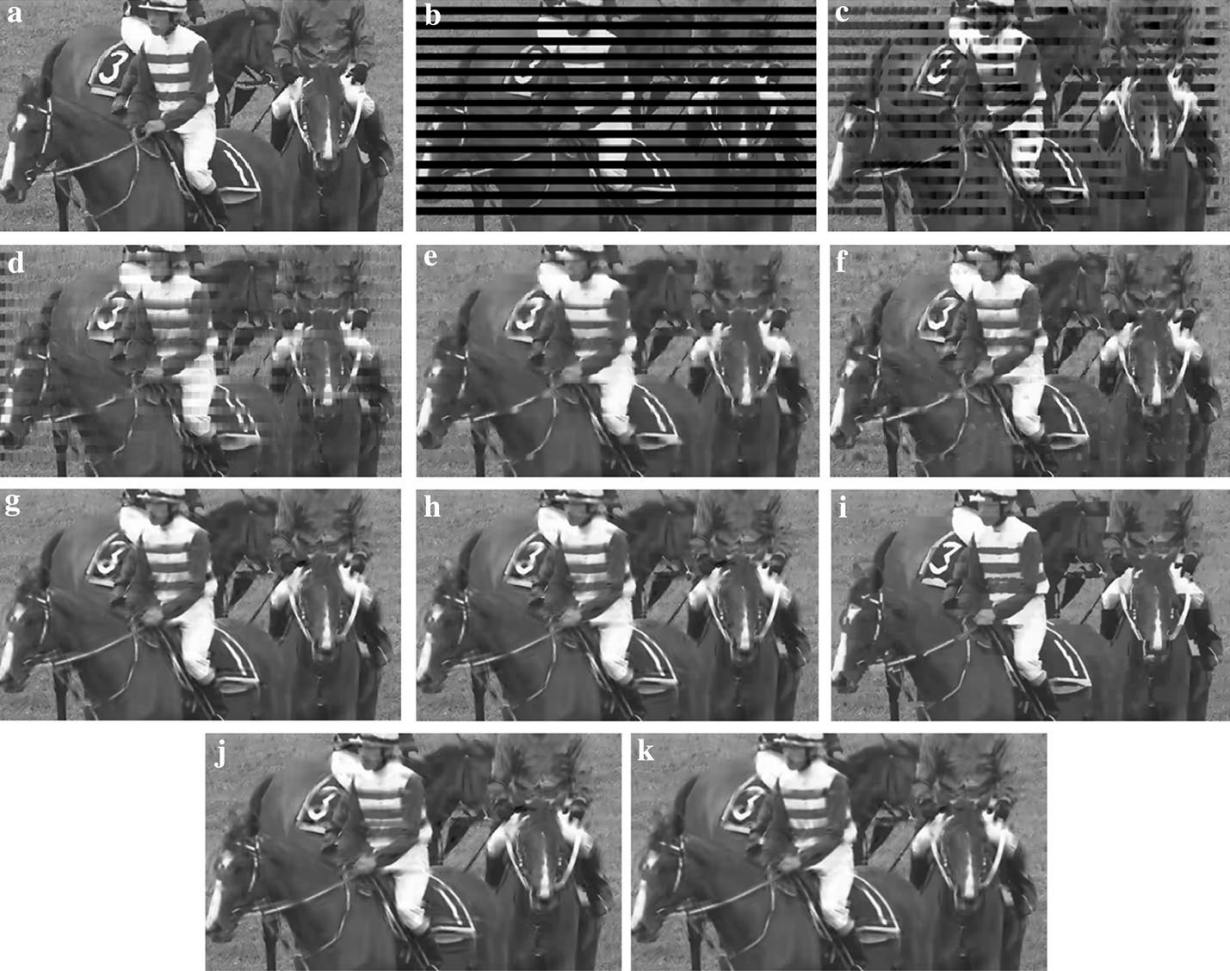
Subjective quality comparisons of different SEC algorithms for *RaceHorses*, 1st intra-frame in QP = 37. **a** The intra-coded frame, **b** the corrupted frame, **c** CAEC, **d** BI, **e** KMMSE, **f** MRF, **g** OAT, **h** HEISI, **i** OALP, **j** XFSE, **k** ESFE

Figures 7 and 8 further show the subjective quality comparisons of reconstructed images by different SEC algorithms, where 1st intra-frame of *Foreman* is encoded with QP = 22, and 1st intra-frame of *RaceHorses* is encoded with QP = 37. As illustrated in these figures, our ESFE algorithm produces the most visually pleasant results among all comparative methods. Although the OALP sometimes gives relatively sharp boundary, it also produces some misleading artificial transition when incorrectly estimating the direction of contours. From these figures in Fig. 7, we can observe that the continuity of hat boundary of *Foreman* is broken when using HEISI, OALP, and KMMSE, while some dominant edges have been reconstructed gracefully by the proposed ESFE algorithm. It is evident that in all SEC algorithms, the proposed ESFE algorithm is the only one that completely recovers the nose of the *Foreman*. From these figures in Fig. 8, it is easy to find that the edges across the regions of consecutive block loss cannot be well recovered with the other SEC methods, and the reconstructed image of the proposed ESFE algorithm is visually more plausible and coherent, such as the edge along saddle and the hat of the rider.

### Run-time comparison

The proposed ESFE algorithm achieves the best average PSNR and MS-SSIM for all test images. To investigate the relative complexity of different SEC algorithms, six images with different sizes (1st intra-frame of *Akiyo* (176 × 144), *Aerial* (256 × 256), 1st intra-frame of *BlowingBubbles* (416 × 240), 1st intra-frame of *Foreman* (352 × 288), *Lena* (512 × 512), 1st intra-frame of *RaceHorses* (832 × 480), *Man* (1024 × 1024)) are tested. By repeating each method 10 times, the average run-time is presented in Table 3, where the run-time is obtained by MATLAB 2012a implementations on Intel Q8200 @ 2.33 GHz CPU and 4 GB memory.

**Table 3:** Average run-time (seconds) of different SEC algorithms

	CAEC	BI	KMMSE	MRF	OAI	HEISI	OALP	XFSE	ESFE
*Akiyo* (176 × 144)	1.57	0.38	349.9	1.95	20.1	96.3	33.5	16.7	17.71
*Aerial* (256 × 256)	8.1	0.72	904.3	4.9	55.3	245.6	91.2	44.3	47.42
*BlowingBubbles* (416 × 240)	9.9	0.97	1503.1	8.2	93.5	405.4	159.3	70.1	75.7
*Foreman* (352 × 288)	6.8	0.91	1437.2	7.9	88.5	393.3	141.9	68.2	74.3
*Lena* (512 × 512)	20.3	1.89	3952.4	21.4	248.2	1004.7	367.3	182.1	200.3
*RaceHorses* (832 × 480)	46.7	2.73	6022.3	33.1	386.3	1529.6	549.1	290.2	322.1
*Man* (1024 × 1024)	131.4	6.71	16293.2	90.9	1062.8	4036.7	1347.2	763.3	854.9

Since these SEC algorithms don’t use any special function, their MATLAB implementations may provide a certain reference for the complexity comparison. From Table 3, it can be seen that the proposed ESFE algorithm is much faster than the recent HEISI and KMMSE algorithms, and it also outperforms OALP when dealing with consecutive block loss. Due to the lack of horizontal correlation, KMMSE has to run a large amount of invalid iterative processes to achieve a convergence result. Although our algorithm requires longer run-time than some methods (e.g., MRF and BI), its advantage is obvious in terms of objective and subjective quality evaluations. Compared with XFSE, simulation results reveal that the proposed ESFE algorithm significantly improves the recovery quality, and increases only a small complexity overhead. Our ESFE algorithm strikes a good balance between the computational complexity and recovery quality.

## Conclusions

To combat the consecutive block loss in transmitted images, this paper presented an effective SEC algorithm by selectively incorporating the edge synthesis into the signal extrapolation architecture. During the iterative approximation, the concealment performance can be improved by using structural information. The edge synthesis via Hough transformation can systematically connect the discontinuous edges before signal extrapolation. With the use of Canny detection and Hough transformation, a contour reconstruction is carried out on degraded bocks and utilized to split the missing blocks into separate regions for signal extrapolation. For consecutive block loss, the proposed ESFE algorithm can reconstruct the dominant structures without high-level semantic knowledge, and thus obtain better subjective and objective quality with a marginal additional computational cost. If object-based edge synthesis or parallel acceleration are available in the future, the performance of the ESFE algorithm will be further improved.
